# Look Before You Leap: What Are the Obstacles to Risk Calculation in the Equestrian Sport of Eventing?

**DOI:** 10.3390/ani6020013

**Published:** 2016-02-16

**Authors:** Denzil O’Brien

**Affiliations:** South Australian Spinal Cord Injury Research Centre, Hampstead Rehabilitation Centre, 207 Hampstead Rd., Northfield, SA 5085, Australia; denzil.obrien@optusnet.com.au; Tel.: +61-883-980-367

**Keywords:** horses, eventing, risk, falls, injury, riders, human−animal relationships, human−horse relationships

## Abstract

**Simple Summary:**

This paper examines a number of methods for calculating injury risk for riders in the equestrian sport of eventing, and suggests that the primary locus of risk is the action of the horse jumping, and the jump itself. The paper argues that risk calculation should therefore focus first on this locus.

**Abstract:**

All horse-riding is risky. In competitive horse sports, eventing is considered the riskiest, and is often characterised as very dangerous. But based on what data? There has been considerable research on the risks and unwanted outcomes of horse-riding in general, and on particular subsets of horse-riding such as eventing. However, there can be problems in accessing accurate, comprehensive and comparable data on such outcomes, and in using different calculation methods which cannot compare like with like. This paper critically examines a number of risk calculation methods used in estimating risk for riders in eventing, including one method which calculates risk based on hours spent in the activity and in one case concludes that eventing is more dangerous than motorcycle racing. This paper argues that the primary locus of risk for both riders and horses is the jump itself, and the action of the horse jumping. The paper proposes that risk calculation in eventing should therefore concentrate primarily on this locus, and suggests that eventing is unlikely to be more dangerous than motorcycle racing. The paper proposes avenues for further research to reduce the likelihood and consequences of rider and horse falls at jumps.

## 1. Background

Equestrian sport is unique. It involves a relationship between two beings, one of which is not human [1]. The horse is a large four-legged prey animal whose successful evolution has resulted from its strong flight instincts [2,3,4]. Humans have sought to use and control horses for thousands of years [5], and over time have extended these uses and controls from simply riding and harnessing horses to involving them in a range of challenging activities: chariot-racing, bull-fighting, thoroughbred racing, jumps racing and buck-jumping, to name just a few.

Eventing is the horse sport usually characterised as the most dangerous of all the modern mainstream equestrian sports [6,7,8]. It is often described as the triathlon for horses [1,8,9], the ultimate test of horse and rider, based on a military tradition. In fact, the French name of the sport is *concours complet*: the complete contest. Eventing demands equine attributes similar to those of a warhorse: obedience, agility and grace on parade (represented by the dressage phase), courage, strength, fitness and speed in battle (the cross-country phase), and a level of fitness and effective recovery which will allow resumption of normal duties immediately after battle (the show-jumping phase) [1,9,10,11].

Eventing is one of the more difficult sports to explain to the uninitiated [10]. First, it has several different names: eventing, horse trials, three-day eventing, and one-day eventing. In this paper, the sport is referred to as eventing. A full explanation of the sport, its scoring and its rules can be found on the website of the Fédération Equestre International (FEI), the peak body for equestrian sports [9].

In the cross-country phase of the sport, horses gallop across open country on a predetermined course, jumping big obstacles within set time limits, incurring penalties for completing too quickly or too slowly. The obstacles may include walls, steps, jumps into and out of lakes and creeks, almost vertical slides, palisades, tables, ditches with fences set within them, banks, keyholes, log piles, sunken roads, and jumps made out of everyday objects such as the flatbeds of trucks, or large carved animals, and often a combination of several of these individual elements [9,10].

The cross-country phase of eventing is generally viewed as the riskiest for both horse and rider [12,13]. These risks include the full range of unwanted outcomes one might expect from an activity which involves riding a horse at speed over fixed obstacles: death, head and brain injury, spinal injury, crush injuries and fractures, as well as minor injuries such as sprains, bruising and abrasions [12,13,14,15,16]. The complex inter-species relationship which exists in all horse-related activity [8,17] is taken to extremes in eventing.

However, until the late 1990s there had not been a great deal of evidence-based research on exactly how risky eventing was [12,13]. The horse world’s attention became focused abruptly, however, when in 1999 five eventing riders died in competition in the UK within a few months of each other [18,19,20], and another died in the USA [20]. Five of these six deaths involved the horse somersaulting over the jump, the rider falling forwards with continued momentum after the horse’s forward motion was abruptly stopped, and the horse subsequently landing on top of the rider (known as a rotational horse fall or a somersault fall [21]).

The FEI responded urgently and promptly to these deaths, and instituted wide-ranging reviews into the sport, with rule changes focusing immediately on reducing the possibility of a rotational horse fall. The 2000 report of the International Eventing Safety Committee [22] stated the following:

A fundamental conclusion which pervades every detailed recommendation is that everything should be done to prevent horses falling: this single objective should greatly reduce the chances of riders being seriously injured, as well as significantly improving the safety of competing horses.

*(p. 2)*

Despite this prompt response, an almost continuous stream of rule and format changes, new requirements governing the construction of fences, and changes to the roles of officials, a further 38 eventing riders have died in or after competition between 2000 and the time of writing (October 2015), including two in Pony Club competitions [20,23,24]. At least 27 of these rider deaths resulted from a rotational horse fall [24].

## 2. Defining Risk in Human—Horse Interactions

All interactions with horses are potentially hazardous. Horses are much larger and heavier than humans, while being sentient, sensitive and prone to a well-developed flight instinct as a direct result of their evolution from a prey animal [2,3,4]. They can travel very quickly, and stop and change direction in less than a second; they can bite, kick, crush and squash—and that is before one has even mounted them [25]. Once one is astride, the ambit of hazard widens to include falling from height, often with speed as an additional hazard, as well as rapid changes in momentum and direction [25].

The notion of risk itself involves much discussion about definitions and meaning, and about methods for calculating, evaluating and mitigating risk in any given situation [26,27]. While one way to calculate risk in a particular activity is to count the number of unwanted outcomes such as injuries or deaths associated with the activity, simply enumerating them is not particularly helpful in determining the actual risks involved for participants, or in managing and reducing those risks, since injuries in sport result from a complex interaction of multiple factors and events [28]. Simple analyses such as counting the number of injuries over a period of time ignore many other contributing factors, and may not be useful in developing appropriate risk-management policies [28]. If one accepts that risk is always present when humans interact with horses [1,2,3,4], and if one accepts that these risks can and should be reduced or eliminated, then an appropriate process to achieve this is to first identify the context within which risk occurs [29], then identify the risks, assess them, control them, and review those controls [30]. Through this process, risk can be quantified, and this quantification can then be used to mitigate risk and help to shape sports injury prevention policies [30,31,32,33,34]. However, before this process can be undertaken, one must first be able to accurately identify cases appropriate to the question.

### 2.1. Data on Human—Horse Interactions

By and large, data about human injury and mortality resulting from human−horse interactions are sourced predominantly from hospital separations records, coronial reports, trauma registries, emergency department records, surveillance programs, surveys and literature reviews.

Research into horse-related injuries tends to focus on the following:

- horse-related injuries in general without specifying a particular equestrian sport, using data from sources such as those outlined above: hospital separations data, injury surveillance programs, coronial data, trauma registries and emergency department data collections [7,35,36,37,38,39,40,41,42,43];
- reviews of the broader literature on overall equestrian-related injury [8,15,44];
- measurement of the incidence of specific horse-related injuries such as spinal injury or maxillofacial injuries, again using sources such as coronial data, hospital separations data, trauma registries, and emergency department data collections, but not focusing on a specific equestrian sport [16,45]; and
- measurement of the incidence of specific injuries such as traumatic brain injury which are associated with keynote sports but which may or may not be an outcome of horse sport [46,47,48].

### 2.2. Challenges for the Researcher

There are a number of challenges facing the researcher who seeks accurate and comparable data on overall horse-related injury, let alone injury resulting from specific horse sports such as eventing.

#### 2.2.1. Data Keeping

In Australia, national information on the epidemiology of overall horse-related injury and mortality has until recently been minimal and fragmented. Cripps [37] was able to provide quite detailed information on horse-related deaths and hospitalisations in Australia using national mortality and hospital separation datasets, but was unable to identify the specific type of horse-related activity, the place of occurrence or the mechanism of injury because of limitations in hospitals’ data coding at the time. He reported that in the year 1996–1997, there were 3124 hospitalisations for horse-related injury, and using the Australian Bureau of Statistics mortality unit data collection, identified 410 horse-related deaths between 1979 and 1998. On this basis he estimated an average of just over 20 horse-related deaths a year at that time, and this figure has been consistently cited since.

The 2014 report by Safe Work Australia (SWA) [30] identifies a total of 11,635 hospital admissions for horse-related incidents between 1 July 2008 and 30 June 2011, an average of 3878 a year, with 40% of these occurring during “sports.” However, “trail or general horseback riding” account for 80% of these so-called sports-related injuries, and neither trail riding nor general horseback riding technically qualifies as a sport, based on the definition of sport as “an organised group activity centred on a contest between at least two parties” [49]. The SWA report also identifies 98 horse-related deaths between July 2000 and June 2012, an average of just over eight per year. Seventy-four per cent of these deaths involved a fall from a horse. However, these data also do not reveal whether the fatality occurred during sport (other than horse-racing), and there is no information on mechanism of injury.

The 2014 report from the Australian Skills Quality Authority (ASQA) [50], initiated after the horse-related death of a young trainee jillaroo in 2009, reports 1,568 hospitalisations resulting from equestrian activities for the 12 months 2011–2012, markedly lower than the average annual number reported by Cripps [37] or the SWA report [30]. ASQA also accessed recent data from the National Coronial Information System, identifying 132 horse-related fatalities between 1 July 2000 and 31 December 2013, an average of just under eight per year. This average is comparable to the findings of the SWA report, but both are less than half the average reported by Cripps [37]. Further, the SWA report points out that 34 horse-related deaths occurred in the 18-month period between 1 July 2012 and 31 December 2013, highlighting the problems which may arise from comparing averages rather than rates. Watt and Finch [45] comment on the difficulties in interpreting and comparing published data on injury in general, because of non-standardised data collection and/or analysis methods. They emphasise that such difficulties are exacerbated in analysis of sport-related injury because of lack of consensus on appropriate definitions. Such lack of consensus is also evident in the 2014 Safe Work Australia report [30].

#### 2.2.2. Data Capture

This leads to a further challenge facing researchers in the area of general horse-related injury: the very wide scope of activities revolving around horses which militates against accurate coding of horse-related injury. “Horse-related activity” includes leading, grooming, feeding or just being with a horse; riding for fun, on a road or in a paddock; going to a competition on the weekend; mustering cattle or sheep; loading and unloading a horse from a float or truck; shoeing or trimming its hooves; training a horse for a specific purpose or competition, including dressage, eventing, show-jumping, endurance riding, polocrosse, driving, vaulting or camp-drafting; competing in one of these sports; horse racing; Western riding; breeding and raising horses; and so on. It is highly unlikely that any hospital injury coding system will capture all of these activities. The World Health Organization’s International Classification of Diseases (ICD) is the most widely used hospital coding system, and in many Western countries, the only coding system in use. The version used in Australia, the ICD-10-AM, includes reasonably comprehensive activity codes, but they do not differentiate between sport and recreation activities, or between professional sport and backyard games [43]. Activity codes relating to sport and recreation are often missing in hospital separation records and as a result there is significant underestimation of the number of cases involved [44]. Even with recent revisions, which allow more codes to describe a specific mechanism and circumstance of injury, there is still consistent allocation of external causes and circumstances of injury to “other” and “unspecified,” which may lead to considerable under-reporting [45]. The ICD-10-AM is not structured to permit the fine-grained coding protocols which will generate accurate information on which specific horse-related activity has resulted in the injury, precisely what injury mechanism has caused it, in which precise location the injury occurred, or which circumstances caused it. This means that it is difficult and indeed unusual for research to compare like with like, since often the specific type of horse-related activity cannot be accurately identified. Such difficulties are not unique to horse-related activities: similar challenges were identified by researchers seeking accurate case records for snow sport injury in New Zealand [51].

#### 2.2.3. Non-Nuanced Research

Yet another challenge in accessing accurate and useful data is that researchers may themselves assess horse-related risk based on possibly inadvertent characterisation of horses, their size and their speed. Researchers may conflate data from the full range of horse-related activity, from recreational horse-riding to competitive equestrian sport, probably because of the difficulties outlined above in accessing specific activity-related data, and also because the researchers themselves may not be personally familiar with the range of horse characteristics and behaviour, nor of individual horse sports. Thus, for example, in a much-cited literature review of equestrian sport-related injury [15], horses are described as weighing “an average of 1500 lbs” (680 kg) and travelling “at up to 40 mph” (64 kph). Given that horses come in all sizes, from the tiny (about 18 inches or 44 cm) to the enormous (about 82 inches or over 2 m) [52,53], average weight is not a particularly useful characterisation when measuring risk for riders.

Similarly, citing the top speed of horses when assessing risk is also not useful [15]. While horses *can* travel at up to 40 mph (64 kph) and more, this is unusual and cannot be sustained for any length of time. Such speed is restricted to specialist conditions such as thoroughbred sprint racing, quarter-horse racing, polo, and perhaps barrel racing, in which explosive bursts of speed are inherent, rather than recreational riding or competitive equestrian sport. For example, the fastest recorded race speed for a winner over 402 m (approximately a quarter of a mile) is 70.76 kph, over a period of 20.57 s [54], and the fastest recorded race speed for a winner of the Melbourne Cup, one of the world’s premier 3200-m races, is 58.32 kph over 3 min and 16.3 s [55]. Citing the average speed from these two examples—64.54 kph—is not useful in measuring risk for riders, since neither recreational nor eventing riders would gallop at such speeds. Indeed, in either a recreational or a competitive sport setting, it is not so much the speed of the horse which contributes to the likelihood of a rider fall, but a sudden acceleration or deceleration, combined with surprise, which will result in a rider falling off (such as the horse breasting a fence and catapulting the rider forwards) [56]. In fact, slower horse speed in eventing may contribute to the risk of a rotational fall resulting in a horse landing on a rider [57].

## 3. Defining Risk in the Sport of Eventing

Given the challenges outlined above in accessing data on general horse-related human injury and fatality, it is not surprising that these challenges are greatly increased when one seeks data specifically related to eventing, and eventing-related injury and death.

### 3.1. Data on Eventing and Injury

One of the major issues for data collection in the sport of eventing is that unless a rider suffers a serious injury requiring hospital admission, medical attention at a trauma centre, or at a hospital emergency department, the injury will be unreported, and will not be captured in the current statistical collection processes.

Specifically in relation to eventing, while the possibility of catastrophic injury or death certainly exists, the great majority of injuries are minor [58]. In an Australian national surveillance project collecting data on rider and horse injuries in eventing from 2002 to 2006 [21], almost all riders who responded to questionnaires about injuries incurred in their cross-country falls characterised their injuries as minor, even while a few of these also reported that they sought medical attention later for serious injuries such as concussion and fractures. One rider responded in the negative to the first question which asked whether they had been injured in their recent fall, and then later in the questionnaire reported that he had later sought medical attention for concussion and broken ribs. Some reports of concussion and fractures were clearly self-diagnoses, since the respondents did not report that they had then gone on to seek medical attention. It is possible that this mischaracterisation of the serious nature of the riders’ injuries is a reflection of the complex inter-species relationship which exists between horse and rider, in which eventing riders express more concern for their horses’ safety than for their own [59]. In any case, the project did reveal a tendency among eventing riders to underestimate the risks involved in the sport, and to under-report any injuries. The surveillance project collected information on 1732 rider falls, in which 374 riders reported at least one injury, ranging from abrasions and bruises through dislocations to concussion and fractures. Because multiple responses were possible, it is not feasible to quantify the exact number of individual injuries. However, of the 1732 falls reported, only 129 were categorised as resulting in serious injury, being fracture, concussion or loss of consciousness. Given that only 900 of the possible 1732 riders who fell completed their questionnaires, it is likely that a similar proportion of riders who did not return their questionnaires were also injured.

### 3.2. Challenges for the Researcher

#### Data Capture

As mentioned above, hospital admission coding systems are not capable of capturing accurate data about general horse-related injury [43,44]. Data collection in the sport of eventing is further complicated by the fact that until relatively recently (2008), a rider who fell during the cross-country phase could remount and continue, even if they were injured [60,61]. Many records of eventing-related injuries were consequently lost to the event organisers and ultimately to data collectors, because they were not reported. In 2008 the rule was changed so that even a simple, non-injurious rider fall at a jump on the cross-country course would result in elimination, and in 2009 this was extended to any fall on course, jump related or not [62]. During the scope of the Australian national surveillance project [21], riders were able to remount and continue, and the project recorded several instances of riders who had incurred quite serious injuries such as concussion or fractures, which later required medical attention or even hospital admission, but who remounted and continued the competition.

Famously, two Australian eventing riders won Olympic gold medals while competing with serious injuries: Bill Roycroft fell during the cross-country phase at the Rome Olympic Games in 1960, suffering multiple injuries including a broken collarbone and severe concussion. He remounted and finished the course, and was then airlifted to hospital. The following day he checked himself out of hospital in order to compete in the show-jumping phase, as the Australian team was down to only two combinations, and needed three to qualify for the medal. He competed with his arm in a sling. Gillian Rolton fell when her horse slipped on grass at the Atlanta Olympic Games and she fractured her collarbone and some ribs. She remounted and continued, and fell again at a water complex as she was unable to use her left arm at all to control her horse. She again remounted and completed the course, successfully jumping another 15 obstacles, riding one-handed. She refused painkillers in case she was required to ride the following day in the show-jumping phase, but fortunately was not required, and was able to get the medical attention she needed. Her heroic gesture won her a second gold medal. Under the current rules, both these competitors would have been eliminated [9].

Even at the present time, it is difficult at the sport-based level to capture all injury records from the sport of eventing. While the FEI requires comprehensive injury reporting from their international event officials and uses these data for their annual reporting on injury rates and severity [58], the organisation concedes that in the past, injury reports were completed by fence judges, based on their own opinion of the severity of the injury incurred [58]. At a national level in Australia at least, there does not yet appear to be any consolidated data which researchers can access. The Australian peak national body, Equestrian Australia (EA), appointed a national safety officer after the FEI Eventing Safety Forum in 2008 [62], but a search of EA’s website using the keywords “eventing safety,” “risk management,” and “eventing falls data” reveals no report from the national safety officer or any other report on the topic, other than a call for expressions of interest in the voluntary position [63]. In addition, in the days when a rider was able to remount and continue, there was strong motivation for riders to conceal injuries so that they could continue in the competition, particularly at the higher levels such as World Championships and Olympic Games. Bill Roycroft and Gillian Rolton both demonstrate that the prospect of an Olympic Gold Medal is a first-class painkiller.

## 4. Quantifying Risk in the Sport of Eventing

Given the background difficulties outlined above in accurate case identification and access to comparable data in the topic of horse-related injury in general, it is not surprising that there is a lack of consensus on how best to quantify and calculate risk in the sport of eventing.

There have been many different approaches to the identification and quantification of risk in eventing outlined in the literature, using many disparate denominators, but usually based on the assumption that rider injury is the unwanted outcome usually employed for calculating risk [12,13,14,15,21]. These approaches have most commonly included:

- Measuring the number of falls as a proportion of the number of participants, assuming that rider and horse/rider falls are the most common cause of rider injury. This process generates a fall incidence rate [21,58].
- Measuring the number of rider injuries as a proportion of the number of participants, again assuming that rider and horse/rider falls are the most common cause of injury. This process generates an injury incidence rate [12,21,58].
- Establishing surveillance and monitoring projects, assuming that more universal capture of data on participants engaged in the actual sport, with details of their actual experience, will provide an opportunity for more fine-grained analysis. Such projects can generate information on a number of factors, including demographic data, situational data, information about the horse/rider combination, and factors contributing to the fall, and usually involve on-ground data collection followed up by questionnaires or surveys [14,21]. This process can generate both fall and injury incidence rates.
- Calculating the number of injuries per hour spent in the activity. This process generates an injury rate per hour [12,14].

### 4.1. Fall Incidence Rate

This particular denominator, which measures the number of rider falls as a proportion of the number of competitors, has been used by the FEI since 2000, when the international body began collecting falls and injury data from all international-level events held under its aegis. This denominator is useful, in that the assessment of risk in eventing is based on the assumption that rider injuries occur mainly as a result of a rider fall, and so calculating the rate of rider falls can provide an insight into the likelihood of rider injury.

The most recent data from the FEI [58], reporting on all competition years from 2005 to 2014, reveal a total of 8556 rider falls from 152,821 starters, a rate of one fall for every 18 starters. These figures include riders whose fall did not involve a jump—*i.e*., falls “on the flat” (*n* = 538).

The Australian national surveillance project [21] reported a rate of one fall for every 34 starters (1732 falls from 58,557 starters) and similarly included falls on the flat, which were not differentiated from falls at fences, because at that time falls on the flat were not penalised and as such were often not even recorded. The authors acknowledged that the figure of 1732 rider falls was very likely an underestimate because of problems with data capture.

These two data sources, widely different in scope and number, reveal a marked difference in rate, with the FEI’s calculation being approximately twice that of the Australian national surveillance project.

### 4.2. Injury Incidence Rate

This denominator, which measures the number of rider injuries as a proportion of the number of competitors, was used in a yardstick analysis by Dr Bruce Paix, an Australian anaesthetist and trauma and recovery specialist who was personally involved in eventing and was the medical officer in attendance at 35 events held in South Australia between 1990 and 1998, about 10% of all such events (including Pony Club events) held in that state during that time. He published his findings [12] to considerable publicity. Using data from the events at which he officiated, involving 4220 competitors, with 37 injured riders, he first estimated the injury incidence rate per competitor at 0.88%. He then compared this incidence rate with other published injury incidence data from other sports, specifically a report which estimated the injury incidence rate for motor racing (both car and motorcycle) participants at England’s Brands Hatch circuit as 0.24% per motorcycle racing competitor [64]. Paix consequently arrived at the conclusion that eventing was more dangerous than motorcycle racing, a conclusion which attracted a great deal of attention and one which has since been consistently cited in the injury literature (55 citations according to Google Scholar at the time of writing).

In relation to injury severity, Paix found that just over 70% of injured eventing riders (26 out of 37) were referred to hospital, with nearly half (12 out of 26) being admitted. If one were using hospital admission as a denominator for serious injury, this would demonstrate a rate of serious injury: 32% of all injured competitors, and 0.28% per competitor.

The FEI also reports annually [58] on the injury incidence rate per competitor, arriving at a rate of 0.65% per competitor, then further broken down by level of injury rather than level of competition. The FEI combines “serious” and “fatal” injuries when reporting on the numbers of riders injured, and does not define “serious” injury. The 2015 report reveals 311 seriously or fatally injured riders in the period 2005 to 2014, from a total of 8556 falls from 152,821 competitors, a considerably larger dataset than that available to Paix (4220 competitors) [12]. The total number of riders injured (slight and serious/fatal) was 978 for the period, revealing that 32% of injured riders were seriously or fatally injured, identical to Paix’s results [12].

The Australian national surveillance project [21] calculated an injury rate of 0.63% per competitor (58,557 competitors, 374 riders reporting at least one injury), almost the same as the overall rate reported by the FEI. This project also reported 31% with serious injuries such as fractures or concussion (*n* = 119), with only 23 riders reporting that they were admitted to hospital.

These three data sources show remarkably similar results for the number of seriously injured riders as a proportion of all injured riders, with two [21,58] also showing a similar injury incidence rate per competitor (0.65% and 0.63%, respectively). Paix’s finding of an injury incidence rate of 0.88% is noticeably higher. This injury incidence rate denominator is particularly useful, as it can further illuminate the risk of a rider injury in eventing, and is also capable of identifying the risk of serious injury.

### 4.3. Surveillance and Monitoring Projects

This method collects all relevant records within a given time period, and might include the number and level of competitions, the total number of competitors, the number of competitors at each level, the number of reported injuries and/or fatalities, and in the case of eventing, the number of rider and horse falls and the number of jumping efforts in the competition. From such data, an incidence rate can be calculated, again using a number of different denominators such as falls per number of starters and injuries per number of falls. Frequently, surveillance projects combine on-site competition data collection with follow-up surveys and questionnaires.

The Australian national surveillance project [21] was one such surveillance project. As mentioned previously, there were some difficulties in data capture and although there was a high return rate for questionnaires sent to riders known to have had a fall cross-country, overall it was understood that the known number of rider and horse falls was considerably lower than those which actually occurred. However, on the basis of the recorded 1,732 rider and horse/rider falls it was possible to estimate a rate of rider falls at 3 per 100 starters over the 5-year period, 1.2 rider falls per 1000 jumping efforts, and an injury rate of 0.2 per 1000 jumping efforts.

The study by Ekberg and colleagues [14] is a similar national surveillance and monitoring study, capturing injury information from all members of the Swedish Equestrian Federation with eventing as their primary discipline (*n* = 513), in a one-year retrospective study. The survey attracted an eventual return rate of 70% (*n* = 357), collecting information on all traumatic injury events, whether in competition or training. The authors reported that 62.8% of injury events occurred during training, and 37.2% during competition. The study sought to capture the incidence of traumatic injury events measured against hours of activity (see 4.4. below), and did not report on injuries as a proportion of the number of starters.

### 4.4. Calculating Injury Rate against Time Spent “in the Saddle”

Paix [12] uses measurement of time spent “in the saddle” to calculate various levels of risk for horse riders. Specifically for eventing, he uses the average time taken to complete a cross-country course (7.5 min) to calculate an overall injury rate of one per 14 h of cross-country riding in competition, and extrapolates this to calculate that the cross-country phase of eventing is over 70 times more dangerous than horse-riding in general. He further estimated that for riders at “the highest level” (inferred as equivalent in difficulty to Olympic or World Championship competition), their rate of one injury per 5.5 h (and at an incidence of 2.2% per competitor per event) is over 180 times that for all forms of horse-riding combined.

Ekberg and colleagues [14] also calculated the incidence of traumatic injury events using the number of injury events reported (*n* = 143), divided by the total person-time at risk, calculating such incidence as 0.54 injury events/1000 eventing hours for novice riders, and 0.35 injury events/1000 eventing hours for qualified riders.

Extensive searches revealed only these two studies which use the specific denominator of “time in the saddle” and so it is difficult to comment on the usefulness of such a denominator. It is certainly worth further exploration, as it is probable that time in the saddle contributes to overall risk in the sport, simply through exposure over time.

All these various approaches assist in illuminating the scope of risk for riders in a sport already well recognised for being “dangerous” [8,12,17]. From these different approaches, it is possible to identify a range of different ways in which to calculate risk for riders in eventing, and these are difficult to assess against each other, employing as they do completely different denominators. There are implications for equestrian sports’ governing bodies whose responsibilities include making the sport as safe as possible, since such studies individually may not prove overly helpful in formulating an overall risk assessment, risk management and risk reduction strategy. Furthermore, labelling a sport as “more dangerous” or “the most dangerous” may have considerable and unintended impacts on participation, and so it is important that any such labels are applied only when the data support them. As outlined above, much of the published research on risk in eventing uses different denominators for calculation, and often compares eventing with other sports which are quite different. For example, if injury risk in eventing is associated primarily with the horse or the rider falling [12,21,22], and that risk is concentrated around the jump itself, and the action of the horse jumping [12,21,22,24,57,58,65], then risk cannot be said to be equally distributed around the cross-country course. In motorcycle racing, for example, risk of injury from falling off the motorcycle would seem to be more equally distributed around the whole racecourse since motorcycle racing involves one continuous action (going as fast as possible) around a relatively uniform track. On the face of it, motorcycle racers are at risk of falling and injuring themselves at almost every minute of a race. Another different aspect of eventing is that the course itself is often over undulating ground, with twists and turns, and frequent changes of pace as riders approach a jump, negotiate the jump, and then cover the ground to the next jump. Thus, a comparison between eventing and motorcycle racing does not seem appropriate in calculating risk for participants.

Table 1 summarises the findings from a very small sample of published work on the risks in eventing, showing the different denominators used in the calculations. It demonstrates the difficulties facing researchers and policy makers who may wish to identify a useful and standard baseline (*i.e.*, the same unit of measurement) for calculation of risk, when no such baseline exists.

**Table 1:** Reports of Fall and injury rates in eventing, 1990–2014.

Denominator	FEI [58] (2005–2014) *n* = 152,821	O’Brien and Cripps [21] (2002–2006) *n* = 58,557	Paix [12] (1990–1998) *n* = 4220	Ekberg *et al.* [14] (2007) *n* = 357
Fall rate per number of starters	1/18 starters	1/34 starters	Not reported	Not reported
Injury rate per number of starters	Slight injury: 1/229 Serious injury: 1/506 Fatal: 1/16,980	Overall rate: 1/156 Serious injury: not reported Fatal injury: not reported	Not reported	Not reported
Injury incidence per starter	Slight and serious injury: 0.65% Fatal: 0.00061%	Overall rate: 0.63% Serious and fatal: Not reported	Overall rate: 0.88% Serious injury: 0.28% Fatal: not reported	Not reported
Injury rate per eventing hour	Not reported	Not reported	1/14 eventing hours overall, 1/5.5 eventing hours “at the highest level“ of competition	0.54 injury events/1,000 eventing hours (novice riders) 0.35 injury events/1,000 eventing hours (qualified riders)
Fall rate per number of jumping efforts	Not reported	1.2 falls/1,000 jumping efforts	Not reported	Not reported
Injury rate per number of jumping efforts	Serious injury: 1/15,687 jumping efforts	Overall: 0.2/1,000 jumping efforts	Not reported	Not reported

## 5. Where Is the Locus of Risk?

While acknowledging that all these approaches can provide valuable information, it may be more useful to explore where the *locus* of risk is in eventing.

Most observers agree that the primary locus of risk in eventing is during the cross-country phase of the sport, where speed and jumping combine, rather than in the dressage or show jumping phases [1,10,57,65]. But this is too general a field from which to identify a precise locus of risk. Multiple factors may combine to produce a fall event which results in a rider’s injury or death: the individual experience of the rider and the horse, and their experience as a combination; the fitness of the rider and the horse; the weather; the light; the ground conditions; the approach to the jump; the capacity of the rider and the horse to judge distance accurately; the skill of the course designer and the jumps builder; and sometimes just plain luck. In a single accident event, it will always be very difficult to exclude any of these contributing factors, or indeed to accurately measure the contribution of each variable and their interactions (clustered data). Sophisticated multilevel statistical analysis such as hierarchical linear modelling is needed in clustered data, and is increasingly being used in analysis of other “risky” sports such as jumps racing [66,67,68].

However, what almost all rider and horse falls in eventing have in common are: the jump itself, the action of the horse jumping, the consequences of the horse jumping, or its failure to jump. Paix’s much-cited article [12] states that “most of” the 37 rider injuries which occurred during the period of study occurred as a result of the rider falling off the horse, or from horse and rider both falling, mostly while jumping an obstacle. No precise numbers are reported, however. The FEI reports that of the 8556 rider falls recorded between 2005 and 2014, 94% of them occurred at a jump [58]. A study by Stachurska and colleagues [69], in identifying risk factors associated with falls cross-country, also focused on the jumps themselves, pointing to factors such as successive elements of combinations, narrow jumps, brush-type jumps, and jumps with alternative routes, across all levels of competition, from novice (one star) to Olympic and World Championship (four star) level. Other studies [57,65] have identified additional jump-related risks associated with jumps with a drop landing and jumps with approaches out of water, as well as riders knowing they were in the lead after dressage, and riders who received tuition. The most recent research commissioned by the FEI has determined that other jump-related factors increase the likelihood of a horse fall: corner fences, square spreads, upright post and rails, jumps into or out of water, downhill terrain, and some combinations of these factors [70].

Mechanically, falls of riders and horses in eventing are usually the result of a sudden loss of the forward momentum of the horse and the continuation of the forward momentum of the rider [71,72]. As mentioned previously, horses can fall “on the flat” during eventing, but compared to the proportion of falls which occur at jumps, falls on the flat do not present the same degree of “danger” for riders [58]. There are at least six scenarios for a fall of a rider during a cross-country course, including:

- away from an obstacle (that is, “on the flat”): the horse slips and falls and the rider continues onwards or sideways, or—rarely—lands under the falling horse;
- the horse refuses to jump, stopping in front of the obstacle, and the rider continues onwards or sideways;
- the horse attempts to clear the jump but hits it, the horse’s forward momentum ceases abruptly and the rider again continues onwards;
- the horse fails to negotiate the jump successfully and the rider is caught up in the ensuing chaos;
- the horse stumbles or falls on landing after jumping, and the rider again continues onwards or is trapped underneath the fallen horse; or
- the horse hits the jump with its chest or front legs, having failed to clear the jump, and its forward momentum carries its body onwards and forwards as it somersaults over the jump. The rider, propelled ahead of the horse’s momentum, lands in in the vicinity of the place where the horse is itself going to land shortly thereafter.

The author, with help from the aforementioned Dr Bruce Paix, has identified and collected information on 59 confirmed rider deaths in eventing since 1993, across all levels of the sport, from Pony Club to Regional Championship [24] (see Appendix Table A1). The collection began in conjunction with the Australian national surveillance project [21], and used search phrases and terms including “rider death eventing,” “cross-country death,” “eventing deaths,” and “rider deaths.” The data collection is based on online articles from recognised newspapers and magazines (see, for example, [18,19]), often followed up by on-line newspaper reports of coronial findings. The fatalities involved 25 males and 34 females ranging in age from 12 to 64, with a median age of 32. There were fatalities at 15 international (FEI)-level competitions, 34 national-level competitions and three Pony Club competitions. In seven cases, there was insufficient information to accurately determine whether the competition was at the international or national level. In 32 cases, the number of the jump is identified, with a range of jump number 2 to jump number 26, with a median of jump number 10. However, there are 25 cases in which the jump number is either unknown or is not adequately identified (for example, “last,” “second last,” “halfway through”). Since the number of jumps and jumping efforts in any one course may vary depending on the level of competition, and within the maximum and minimum number of jumping efforts required at that level of competition [9], it is not possible to extrapolate any meaningful information from these data.

**Figure 1:**
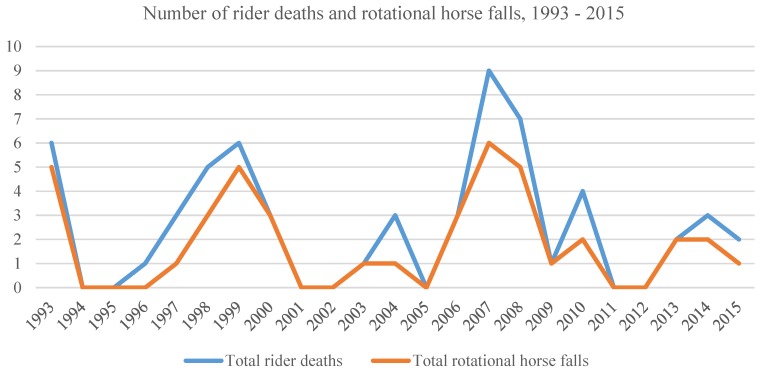
Rotational horse falls and rider fatalities 1993 to 2015.

Figure 1 shows the total number of known rider deaths between 1993 and 2015 (*n* = 59), and those known to have been the result of a rotational horse fall (*n* = 41). In eight cases, there is no verifiable information on whether the horse fall was rotational or not.

While risk in eventing is unevenly distributed, there is a very strong demonstrated bias towards jumping efforts (that is, the number of times a horse is required to jump an obstacle in any one eventing course) [21,58]. It would suggest, therefore, that the primary locus of risk is the jump itself, and the action of the horse jumping, and that, as the Hartington Report stated, the best chance of reducing risk for riders and horses in eventing consists of reducing the chance of the horse falling [22]. Since the horse is most likely to fall at a jump, this suggests that the greatest risk occurs at the jump. In this case, the focus of future research should be on this locus of risk, and its relationship with the other factors which contribute to risk in the sport. Calculations of risk in eventing should concentrate first on measuring the number of rider and horse/rider falls and the number of rider injuries in relation to the number of jumping efforts performed by the horses.

The FEI has led the way in developing changes to the infrastructure of the sport in order to reduce risk for riders and horses, and these changes revolve primarily around the jump, its construction, and the rules governing how it can be jumped [73]. First, after the sudden cluster of six rider deaths in 1999, five of them resulting from rotational horse falls, the FEI immediately changed the rules governing how a rider might re-present a horse to a jump if it had refused, or not approached the jump correctly. Until this time, as long as the horse was not deemed to have stepped backwards, the stop was not considered a refusal, and therefore incurred no penalties. The rider could simply ask the horse to jump from a virtual standstill after an initial failure to jump, greatly increasing the likelihood of the horse breasting the fence because of lack of momentum, dislodging the rider forwards, and then somersaulting over the jump onto the rider on the other side. The rule changes now require a rider to circle away and attempt the jump again, incurring penalties, or opt to take a longer, slower option with alternative obstacles, which will also incur penalties. Secondly, the FEI has overseen and financed the development and introduction of significant technical interventions in jumps construction, with the invention of so-called “frangible pins,” a system which deconstructs the jump when it is subjected to the force exerted by a horse colliding with it (*i.e.*, at the start of what might otherwise be a rotational horse fall). Interestingly, the most recent research from the FEI has found an increased risk for horses falling at fences with frangible pins [70], definitely an unforeseen consequence of a research-based safety innovation, and clearly a focus for further research. The FEI has also supported research and development in the areas of helmet manufacture and body protectors, ensuring that comparable safety standards across all jurisdictions could allow the FEI to mandate the use of particular helmets and body protectors [74]. Thirdly, there have also been changes to the qualification requirements for riders and horses, with the goal of ensuring higher levels of skill before higher levels of competition [73]. In addition, the FEI’s comprehensive data collection from all international and national-level events [58] has provided a base upon which to assess risk with a greater degree of accuracy than simply counting numbers of rider falls and injuries.

## 6. Conclusions

As outlined previously, a multitude of factors interact to result in a fall of rider and/or horse in the sport of eventing, and the risk of such falls is neither evenly distributed nor constant. However, given that more than 90% of these falls happen at the jump itself [58], future research should then focus appropriately. The FEI is already undertaking a major research project on the factors which contribute to falls and injuries, first by examining the role played by specific fence types in horse falls [70], and then concentrating on rider and horse qualifications. However, this paper’s contention that the primary locus of risk is the jump itself should be further tested by analysis of larger datasets than those held in the current FEI database, which includes only data on international-level events. Systems should be established to ensure accurate recording of the circumstances surrounding all falls of riders and/or horses at all competition levels, in every eventing country in the world, including information on as many variables as possible. Such data will provide the basis for future multilevel analyses which may help unravel the many variables at play in such a complex set of clustered data. 

Eventing is an expensive sport for organisers. If further research can support the contention that the primary locus of risk is the jump itself, then this will encourage organisers to concentrate their limited resources on jump-related interventions in the first place, while not ignoring other interventions.

Other avenues for future research include determining what is an acceptable level of risk in the sport of eventing, and for which group such a level of risk is acceptable. What is an acceptable risk level for the riders? For the horses? For the public? For the organisers? Each group will almost certainly give a different assessment, and these assessments must play a role in arriving at an overall assessment of risk, and an acknowledgement of a degree of acceptance of risk. The acceptability of risk within a specific sport is dependent on the perceptions of the participants [31] and on those of the observers as well. As far as the riders are concerned, one research study [59] suggests that eventers are generally more concerned about injury to their horses than to themselves, and that riders identify the horse itself as the source of risk, at the same time expressing the view that an effective partnership between horse and rider is the best form of risk mitigation. Thus the horse is represented as “both source and saviour of risk” [59].

This paper has not dealt at all with the risks for horses in eventing, but at least 74 horses are known to have died in competition or immediately afterwards, since June 2005, an average of more than seven a year [23]. The fate of eventing horses is not well documented, as their deaths have been rarely reported until relatively recently, and they may very well sustain an injury which requires euthanasia away from the competition. No central record system exists for eventing horses, other than their competition record. In the current climate of strong public antipathy to the perceived cruelty to animals [75,76,77,78], combined with ambivalence about the use of animals for entertainment [61,79,80], the issue of horse fatalities in the sport of eventing will undoubtedly soon attract public attention, as it has in the sport of jumps racing [75,76,78]. The unpublished reports of Harrison and colleagues [67,68] found a total of 113 horse deaths in jumps racing in the Australian State of Victoria between 1995 and 2005, an average of 10 a year. The recent report [78] on Australian jumps racing, conducted now only in Victoria and South Australia, reveals 10 horse deaths in jumps racing between 2012 and 2014, an average of five a year. The fate of thoroughbred racehorses, whether flat racers or jumps racers, has until very recently also been unknown once the horse leaves the racetrack for the last time. In 2015, Racing Victoria Ltd changed its rules to require trainers to notify them of the death of a horse in training [78]. No such requirement currently exists for eventing horses. Accurate data on horse injuries and deaths in the sport of eventing might go some way to addressing concerns about the lives of eventing horses after competition.

Eventing can never be totally risk-free for riders or horses. Risk is inherent to the activity, and those who engage in the sport and those who manage it know and understand this. If risk assessment serves to reduce and minimise risk, then any calculation of risk in eventing in comparison with other activities should first be based on comparing like with like. If risk in eventing is unevenly distributed and not constant, then using comparisons with other activities in which risk is both evenly distributed and relatively constant—such as motorcycle racing—is not likely to throw light on the topic. Although more research needs to be done on the topic, risk calculation based on the number of rider injuries and deaths as a proportion of the number of jumping efforts would indicate that eventing is unlikely to be as dangerous as motorcycle racing. The focus of risk calculation in eventing should be first on the jump, and the action of the horse jumping, and on preventing horses from falling at jumps. It is by focusing on the locus of risk—the jump—that eventing will have a sporting chance.

## Appendix 1.

**Table A1:** Fatalities in eventing, 1993—2015.

Date	Name	Age	M/F	Location	Injury Mechanism	Level	Rotational Horse Fall	Circumstances	Fence #and Type
29/05/1993	Richard Adams	23	M	England (Windsor)	Fall	NAT Advanced 3DE	Yes	Horse hit fence then landed on rider (source: internet)	#14 table
5/06/1993	Malcolm Munro-Kerr	42	M	England (Lowesby)	Hit head on ground	NAT Preliminary	Not reported	Fell. No other information (source: internet)	#19 third element, post and rails
6/06/1993	Verna Hiltebeitel	24	F	USA (Douglassville)	Crush	Not reported	Yes	Horse somersaulted, landed on rider (source: Bruce Paix (BP), internet)	Not reported
16/06/1993	Mark Holliday	23	M	England (Hexham)	Crush	Not reported	Yes	Horse somersaulted, landed on rider (source: internet)	Not reported
18/08/1993	Vanessa Weaver	40	F	England (Tythrop)	Crush	Not reported	Yes	Horse's legs became entangled in rails, rider fell, horse somersaulted, landed on rider (source: internet)	Sloping rails
3/10/1993	Karen Smart	28	F	England (Asgarsby)	Crush	*Pony Club* Hunter Trials	Yes	Horse refused at jump, somersaulted over on top of rider, landed on top of her (source: internet).	#15 Upright of telegraph poles
22/04/1996	Natalie Dunlop	14	F	Australia (Penola)	Head *vs.* tree	NAT Grade III	No	Horse bolted, rider thrown at high speed into tree (Source: BP; internet)	Not reported. Possibly not fence related
18/05/1997	Anna Savage	36	F	Australia (Naracoorte)	Blunt trauma	NAT Open I/M	No	Horse stumbled in ditch, not fence-related (“near end of course”). (Source: BP; personal information)	*Not fence related*, near end of course
6/09/1997	Sam Moore	35	M	England (Blenheim)	Crush	FEI CCI *******	Yes	Horse lacking energy. Horse hit fence (keyhole), rider catapulted, horse somersaulted, landed on rider's chest (source: internet)	#19 keyhole
20/09/1997	Amanda Warrington	29	F	USA (Fair Hill)	Blunt trauma	FEI CCI *******	No	Fell off, major head injuries. Unconscious for several months, died *28/12/1997* (source: internet)	Table
14/03/1998	Linda Riddle	37	F	USA (Trojan)	Not reported	Not reported	Yes	Referred to in Internet forum: "Linda Riddle died when her horse misjudged the bounce … competing advanced." Horse hit the second element and the rider flew over the fence, the horse then jumped on the rider. (source: BP; internet)	Bounce, rails and ditch
22/03/1998	Tasha Khouzam	18	F	Australia (Jupiter Creek)	Crush	NAT Novice	Yes	Horse hit fence, somersaulted over on top of rider (source: BP; internet)	Log fence, 4′ wide
13/04/1998	David Foster	43	M	Ireland (Rathmolyon)	Crush	NAT Advanced	Yes	Horse somersaulted over on top of rider at water jump (source: internet)	Not reported
20/06/1998	Keith Taylor	32	M	USA (Radnor)	Crush	International' (unconfirmed) Level not reported	Not reported	Horse made an error in jumping and fell, landing on rider. (source: BP, internet)	Coffin/ditch
11/10/1998	Roberta Scioscia	43	F	USA (Tryon)	Unknown	NAT Preliminary	Not reported	Death notice in *NYTimes* (online), 11/10/1998. (source: internet)	# 3, Log,
1999 ^†^	Ken Machette	58	M	USA (Trojan)	Crush	NAT Preliminary	Yes	The horse jumped from a standstill, flipped and landed on rider. “Broke Ken in half.“ (source: BP, internet)	Log drop into water
15/05/1999	Peta Beckett	33	F	England (Savernake)	Blunt, crush	NAT Novice	Yes	Horse hit fence (bounce, offset rails), somersaulted over on top of rider (source: internet)	#7 & 8 double (bounce), rails
27/06/1999	Robert Slade	30	M	England (Wilton Park)	Crush	NAT Intermediate	Yes	Attempted bounce fence with insufficient impulsion, horse fell on rider (source: BP, internet)	#17 double (bounce)
22/08/1999	Polly Phillips	30	F	Scotland (Thirlestane Castle)	Crush	International“ (unconfirmed) Scottish Champion-ships	Yes	Horse hit fence hard, somersaulted over on top of rider (source: BP, internet)	#10 Oxer
4/09/1999	Simon Long	38	M	England (Burghley)	Crush	FEI CCI ********	Yes	Horse refused at direct element (water), was immediately turned to attempt the RH option, hit fence with both front legs and somersaulted onto rider. (Source: BP, internet)	#20 jump out of water
26/09/1999	Peter McLean	20	M	England (Somerleyton)	Crush	NAT Intermediate	No	Parallel rail fence, horse stumbled on landing, fell on rider (source: internet)	Oxer
9/04/2000	Mark Myers	32	M	Australia (Fig Tree Pocket)	Crush	NAT Intermediate	Yes	Horse hit second element of bounce, rail into water. Horse somersaulted over onto rider, rider already dead when medical team arrived. (source: internet)	#10 (approx)
16/04/2000	Rhonda Mason	35	F	Australia (Camperdown)	Crush	NAT Pre-novice	Yes	“Horse was on the forehand, and galloping downhill, and just didn’t see the fence because its head was too low“ (eyewitness, Internet forum). Horse hit fence, stumbled on landing and rolled onto rider (source: internet)	#15 4-rail roost
30/04/2000	Jemima Johnson	38	F	England (Wilmslow)	Crush	NAT Advanced	Yes	Horse failed to make the height, hit log pile on the rise, somersaulting over onto rider (source: internet)	Not reported
25/08/2003	Samantha Hudson	33	F	England (Moreton-in-Marsh)	Crush	NAT Novice	Yes	Horse hesitated, hit fence (log over ditch) with chest, somersaulting over onto rider (source: internet)	#14
24/07/2004	Cindy Burge	41	F	USA (Rebecca Farm)	Crush	NAT Level unknown	No	Not fence related. Horse slipped on turn, rolled onto rider. *Died 28/7/04* (source: internet)	*Not fence related*
29/08/2004	William Booth	51	M	USA (Senator Bell Farm)	Blunt trauma	NAT Level unknown	No	Horse refused at stone wall, throwing rider into wall (source: internet)	Not reported
5/09/2004	Caroline Pratt	41	F	England (Burghley)	Crush (in water)	FEI CCI ********	Yes	Horse clipped jetty, somersaulted over onto rider in shallow water (source: internet)	#26 (third last)
21/08/2006	Sherelle Duke	28	F	England (Brockenhurst Park)	Crush	NAT Advanced	Yes	Horse hit second element of double, somersaulting over onto rider (source: internet)	Not reported
23/11/2006	Mia Erikkson	17	F	USA (Galway Downs)	Crush (in water)	FEI CCI ******	Yes	Slow rotational fall. Horse fell on her after rotational fall. *Sister Shana died in riding accident in 2003* (source: internet)	#19
8/12/2006	Kim Hyung-Chil	47	M	Qatar (Doha)	Crush	FEI CCI *****	Yes	Horse caught front legs on upper part of jump, somersaulted over onto rider (source: internet, YouTube)	#8
17/02/2007	Amanda Bader	32	F	USA (Ocala)	Blunt trauma	NAT Prelim	No	Fell from her horse, sustained head injury. *Died 28/2/07* (source: internet)	Not reported
14/03/2007	Amelie Cohen	30	F	France (Fontainbleu)	Crush	NAT Novice	Yes	Horse somersaulted at fence and landed on top of rider (source: internet)	#7
18/04/2007	Jo-Anne Williams	34	F	England (Sapey)	Blunt trauma	NAT Novice	No	Rider sustained serious head injury when her horse hit the top of a bench jump (source: internet)	#8
8/05/2007	Julie Silly	17	F	France (Jardy)	Crush	NAT Novice	Yes	Horse fell heavily on rider after rotational fall at “house fence” (source: internet)	#3b
21/07/2007	Elin Stalberg	19	F	Sweden (Bollnas)	Unknown	NAT Intermediate	Yes	Horse somersaulted over fence (corner fence), landed on rider (source: internet)	Third last
31/07/2007	Anke Wolf	40	F	Germany (Neu Wulmstorf)	Crush	NAT Novice	Yes	Horse somersaulted over last fence, landed on top of rider (source: internet)	Last
4/08/2007	Tina Richter-Vietor	32	F	Germany (Schenefeld)	Blunt trauma	FEI CIC ******	No	Horse fell after catching a leg in fence, rider thrown right, horse fell left (source: internet)	#2
2/09/2007	Maia Boutanos	29	F	France (Moulin)	Crush	NAT Novice	Yes	Portable fence moved on impact, fell over, causing horse to fall on the rider (source: internet)	#5b
19/11/2007	Eleanor Brennan	21	F	USA (Ocala)	Crush	FEI CCI ******	Yes	Horse struck fence (table), rolled over onto its neck, trapping rider. Horse died instantly (source: internet)	#18
29/01/2008	Shannon Bloomfield	12	F	England (Malt Hill Farm)	Crush	*Pony Club*	Yes	Horse clipped the jump, and landed on the child, kicking her in the head as it got to its feet (source: internet).	Not reported
6/04/2008	Franz Graf	62	M	Austria (Aspang)	Crush	NAT Prelim	Yes	Horse hung a foreleg at fence (table) and somersaulted over onto rider (source: internet).	Last
20/04/2008	Karen Rodgers	41	F	Ireland (Ballindenisk)	Crush	NAT Advanced	Yes	Horse fell at fence (water) and landed on rider (source: internet).	Second last
9/08/2008	Emma Jonathan	23	F	England (Hartpury)	Crush	FEI CCI ******	Yes	Horse struck fence and flipped vertically, landing on rider (source: internet)	#19
30/09/2008	Debbie Atkinson	unknown	F	USA (Kentucky)	Not reported	NAT Intermediate	Not reported	'Fell at an intermediate cross-country fence on *2/9/2006*. Rider died 2 years later, *30/9/2008* (source: internet)	Not reported
15/08/2008	Stephen Moore	45	M	Ireland (Antrim)	Not reported	Unknown	Not reported	Fell at event *2* *August 2008*, suffered injuries, discharged from hospital, died of unknown causes. *Twin brother Sam died at Blenheim in 1997* (source: internet)	Not reported
30/10/2008	Jade South	15	F	England (Redmarley)	Crush	*Pony Club*	Yes	Horse hesitated at fence, rider pushed on. Horse hit portable fence with front legs, fence lifted 10" off the ground, horse somersaulted over fence with rider still in the saddle. Landed on top of rider (source: internet).	Not reported
26/04/2009	Ian Olding	47	M	England (Belton)	Crush	NAT Advanced	Yes	Rotational fall at table element of combination, horse landed on rider (source: internet)	#13, halfway
18/03/2010	Dirk Grouwels	48	M	Belgium (Zutendaal)	Probably crush, as rider was killed instantly after horse somersaulted.	NAT Possibly unaffiliated event	Yes	Horse refused at first part of combination, rider insisted and the horse somersaulted over the fence and landed on rider (source: internet).	Not reported
24/05/2010	Elena Timonona	15	F	Russia (Krasny Voskhod)	Crush	NAT CNC *****	Yes	Rotational fall at an upright fence. YouTube video shows horse failed to lift front legs, went straight over the fence and landed fully on rider. Rider does not move after fall. Horse trotted away (source: YouTube, internet)	#16
14/09/2010	Robin Donaldson	64	M	England (Beckwithshaw)	Landed on his head	NAT BE100 (pre-novice)	No	Horse straddled a fence and rider fell over her neck and landed on his head. Fell on *21* *August 2010*, died *14 September 2010* (source: internet).	Not reported
18/09/2010	Sebastian Steiner	22	M	Italy (Montelibretti)	Not reported	FEI CCI *******	Not reported	No information available	10C (or 11)
2/03/2013	Bruno Bouvier	60	M	Portugal (Barocca d’Alva)	Not reported	FEI CIC ******	Yes	Rotational fall at fence. Horse trod on its own shoe and tripped into brush arrowhead fence, landed on rider (source: internet)	#5 arrowhead
19/08/2013	Tom Gadsby	26	M	UK (Somerford Park)	Crush	FEI CIC *****	Yes	Rotational horse fall at fence, table type, landed on rider (source: internet)	#4B table
14/06/2014	Jordan McDonald	30	M	England (Nunney)	Crush	NAT Novice	Yes	Rotational horse fall, landed on rider “The horse struck the jump with its front legs and slowly rotated across the jump and on to the other side, throwing its rider, Jordan McDonald, to the ground and landing full square on top of him.” (Coroner’s report) (source: horseandhound.co.uk).Coroner found that body protector did not meet British Eventing’s standard (source: internet).	#7a
14/06/2014	Benjamin Winter	25	M	Germany (Luhmühlen)	Crush	FEI CCI ********	Yes	Rotational horse fall at fence 20 (second ride in class), landed on rider (source: internet)	#20 Table
12/11/2014	Guillaume Pucci	28	M	France (Le Pouget)	Fell from horse	FEI CIC ******	Not reported	Fell *12/11/2014, Died 14/6/2015, after being in a medically induced coma since the fall.*(source: horsetalk.co.nz)	#8
14/02/2015	Francisco Seabra	30	M	Spain (Seville)	Not reported	FEI CIC ******	Not reported	Horse and rider fall (source: internet)	#10C 3rd element of water complex
12/04/2015	Sabrina Manganaro	25	F	Italy (Cuceglio)	Fall when horse fell. Possible kick from horse.	NAT Level 3 (equivalent to Preliminary)	Yes	Rotational horse fall. Horse apparently underestimated the size of the fence and fell (source: internet)	#13 “Skinny“ sloping log fence

Notes: **^†^** Exact date unknown; ***, **, ***, ****** The number of stars at FEI level events signifies degree of difficulty, with * being the lowest and **** being the highest.
